# Formation Mechanism and Biomedical Applications of Protease-Manipulated Peptide Assemblies

**DOI:** 10.3389/fbioe.2021.598050

**Published:** 2021-02-26

**Authors:** Tianyue Jiang, Chendan Liu, Xiao Xu, Bingfang He, Ran Mo

**Affiliations:** ^1^School of Pharmaceutical Sciences, Nanjing Tech University, Nanjing, China; ^2^State Key Laboratory of Natural Medicines, Jiangsu Key Laboratory of Drug Discovery for Metabolic Diseases, Center of Advanced Pharmaceuticals and Biomaterials, School of Life Science and Technology, China Pharmaceutical University, Nanjing, China

**Keywords:** protease, self-assembly, mechanism, peptide assemblies, biomedical application

## Abstract

Exploiting enzyme-catalyzed reactions to manipulate molecular assembly has been considered as an attractive bottom-up nanofabrication approach to developing a variety of nano-, micro-, and macroscale structures. Upon enzymatic catalysis, peptides and their derivatives transform to assemblable building blocks that form ordered architecture by non-covalent interactions. The peptide assemblies with unique characteristics have great potential for applications in bionanotechnology and biomedicine. In this mini review, we describe typical mechanisms of the protease-instructed peptide assembly *via* bond-cleaving or bond-forming reactions, and outline biomedical applications of the peptide assemblies, such as drug depot, sustained release, controlled release, gelation-regulated cytotoxicity, and matrix construction.

## Introduction

Bottom-up fabrication is related to a precise control and generation of structures with desired shapes and characteristics starting from smaller dimensions by the self-assembly of atoms or molecules. Bottom-up fabrication approach presents a novel direction to achieve well-defined and functional nano-, micro-, and macroscale structures (Um et al., 2006; Hauser and Zhang, 2010; Chen and Liang, 2012; Yu et al., 2015). Oligopeptides are fascinating backbone of building blocks for self-assembly due to their rich chemistry, ease of synthesis, high biocompatibility, and good biodegradability (Restu et al., 2018; Ma et al., 2020). The peptide assembly is often driven by manifold physical and chemical stimuli, such as temperature (Draper et al., 2017; Lampel et al., 2017), pH (Ghosh et al., 2012), light (Haines et al., 2005), metal ions (Zou et al., 2015), and salinity (Ozbas et al., 2004). Enzyme-mediated reactions as selective biological stimuli, including protease (Toledano et al., 2006; Xu et al., 2014), phosphatase (Tian et al., 2014; Feng et al., 2018; Wang et al., 2018), and carboxylesterases (Li et al., 2015; Zhang et al., 2019), are considered as a preferential approach to induce self-assembly, which has numerous advantages of unique chemo-, regio-, and enantio-selectivity (Sakurai et al., 1988), and mild reaction conditions.

Proteases as a large class of enzymes possess catalytic function to hydrolyze proteins and peptide, which are extensively found in animal offal, plant stems and leaves, fruits, and microorganisms. In the human body, proteases are involved in the occurrence and development of various physiological/pathological activities (Coughlin, 2000; Visse and Nagase, 2003; Lopez-Otin and Bond, 2008), such as food digestion and absorption, blood coagulation, cell differentiation and autolysis, aging, cancer progression, and metastasis. The protease catalysis approach for peptide synthesis and assembly has attracted considerable attentions over the past few decades (Morihara and Oka, 1981; Ulijn et al., 2002). Under the catalysis of protease, the peptides and their derivatives experience biochemical reactions accompanied by structural changes, and subsequently produce nanosized supramolecular building blocks, which arrange spatially and form specific architecture such as nanofibers *via* intermolecular non-covalent interactions. The assembling nanofibers would further undergo transformation in macroscopic aspect. For example, a sol-gel transition leads to formation of a macroscale hydrogel. In this mini review, we describe typical mechanisms of the protease-instructed peptide assembly, and outline biomedical application of the peptide assemblies.

## Protease-Instructed Assembly Mechanism

**FIGURE 1:**
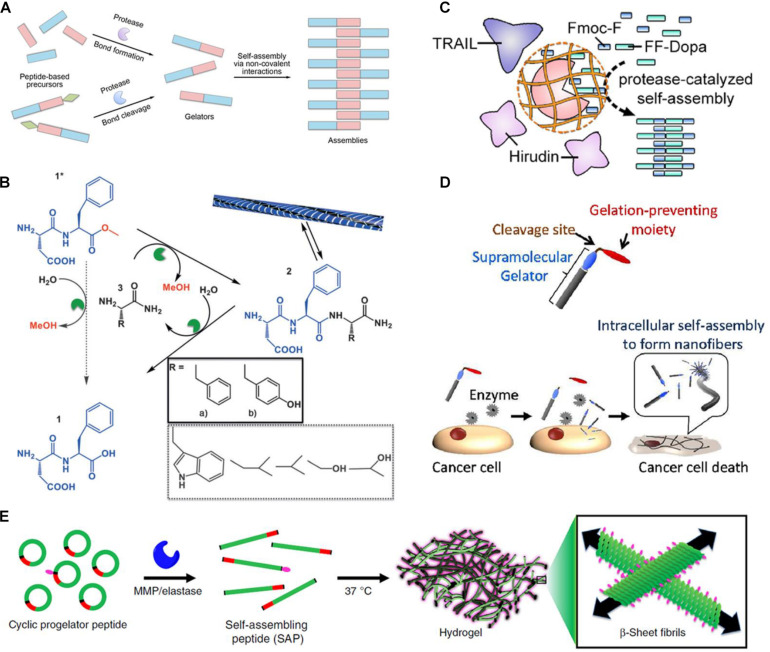
**(A)** Schematic of protease-triggered bond-formation or bond-cleavage reaction followed by self-assembly. **(B)** Transient hydrogel based on the peptide nanofiber assembly triggered by *a*-chymotrypsin under kinetic control (reprinted with permission from *Angew. Chem. Int. Ed. 2015, 54, 8119–8123*). **(C)** Schematic of substrate-selective protease-assisted self-assembly of the peptide hydrogel for protein delivery (reprinted with permission from *Nano Lett. 2017, 17, 7447–7454*). **(D)** Schematic of selective cytotoxicity of endogenous MMP-7-triggered *in situ* formed peptide assemblies against cancer cells (reprinted with permission from *J. Am. Chem. Soc. 2015, 137, 770–775*). **(E)** Schematic of endogenous enzymatic conversion of cyclic peptides into linear peptides for *in situ* hydrogel formation (reprinted with permission from *Nat. Commun. 2019, 10, 1735*).

The mode of the protease-manipulated self-assembly generally involves a combination of covalent and non-covalent interactions. The non-assembling precursors converse into building blocks by protease-catalyzed bond-cleaving or bond-formation reactions, which self-assemble into specific architecture *via* non-covalent interactions mediated by molecular recognition including hydrophobic interaction, hydrogen bonds, π–π stacking, and β-sheet interaction (Loo et al., 2012; Fichman and Gazit, 2014). According to the types of proteases and their specific substrates, both bond cleavage and bond formation are feasible in the protease-manipulated self-assembling systems (Figure 1A). A summary of the commonly used proteases with the corresponding precursors and gelators is listed in Table 1.

**TABLE 1:** Summary of commonly-used proteases with their corresponding precursors and gelators.

Enzymes	Precursors		Gelators	References
MMP-9	FFFFCGLDD		FFFFCG/FFFF	Yang et al., 2009
Thrombin	PEG_4_-_*D*_R_*D*_R_*D*_SP-LTPR-PABA-F_5_Phe-Phe-NH_2_		PABA-F_5_Phe-Phe-NH_2_	Bremmer et al., 2012
Chymotrypsin	PEG_4_-_ D_R_*D*_R_*D*_SP-AAPF-PABA-F_5_Phe-Phe-NH_2_			
Glu-C	PEG_4_-_ D_R_*D*_R_*D*_SP-DAFE-PABA-F_5_Phe-Phe-NH_2_			
Thermolysin	Fmoc-A	FF	Fmoc-AFF	Toledano et al., 2006
	Fmoc-V		Fmoc-VFF	
	Fmoc-L		Fmoc-LFF	
	Fmoc-F		Fmoc-FFF	
Thermolysin	Fmoc-F	FF	Fmoc-FFF	Williams et al., 2009
	Fmoc-L	LL	Fmoc-LLLLL/Fmoc-LLL	
	Fmoc-T	L-OMe	Fmoc-TL-OMe	
	Fmoc-T	F-OMe	Fmoc-TF-OMe	
α-Chymotrypsin	KL-OEt		(KL)*_*x*_*	Qin et al., 2013
α-Chymotrypsin	DF-OMe	F-NH_2_	DFF-NH_2_	Pappas et al., 2015
		Y-NH_2_	DFY-NH_2_	
Thermolysin	Fmoc-T	F-NH_2_	Fmoc-TF-NH_2_	Conte et al., 2018
Chymotrypsin	DF-OMe		DFF-NH_2_	
WQ9-2	Fmoc-F	FF-Dopa	Fmoc-FFF-Dopa	Jiang et al., 2018
MMP-9	PhAc-FFAGLDD		PhAc-FFAGL	Kalafatovic et al., 2016
	GFFLGLDD		GFFLGL	
MMP-7	Palmitoyl-GGGHGPLGLARK		Palmitoyl-GGGHGPLG	Tanaka et al., 2015
MMP-9	NH_2_-^∗^C(KLDL)_3_PLGLAGC^∗^-NH_2 *Cyclic*_		H_2_N-LAGC-C(NH_2_) (KLDL)_3_PLG-OH	Carlini et al., 2019
	NH_2_-^∗^C(KFDF)_3_PLGLAGC^∗^-NH_2 *Cyclic*_		H_2_N-LAGC-C(NH_2_) (KFDF)_3_PLG-OH	
Elastase	NH_2_-^∗^C(KLDL)_3_PLGLAGC^∗^-NH_2 *Cyclic*_		H_2_N-GC-C(NH_2_) (KLDL)_3_PLGLA-OH	
	NH_2_-^∗^C(KFDF)_3_PLGLAGC^∗^-NH_2 *Cyclic*_		H_2_N-GC-C(NH_2_) (KFDF)_3_PLGLA-OH	
Thermolysin	Fmoc-T	L-NH_2_	Fmoc-TL-NH_2_	Wang et al., 2020
	Fmoc-Y		Fmoc-YL-NH_2_	
Thermolysin	Fmoc-L	LL	Fmoc-LLL	Williams et al., 2011

*PABA, *p*-aminobenzoic acid; F_5_Phe, pentafluorophenylalanine; ^∗^represents a disulfide bond between two cysteines.*

### Assembly by Bond-Cleaving Reactions

Protease-catalyzed bond-cleaving reactions prevail in biological processes. A host of proteases with specific catalytic activities in human body have been applied as stimuli to obtain peptide-based building blocks for self-assembly of ordered structures, such as matrix metalloproteinase (MMP), thrombin, and chymotrypsin. For example, MMP can degrade specific amino acid sequences, and each member of the MMP family has certain substrate specificity (Turk et al., 2001; Ratnikov et al., 2014). The non-assembling precursor often consists of building block, hydrophilic moiety, and protease-recognizing linker. The protease-catalyzed hydrolysis of the precursor often leads to removal of hydrophilic moieties and generation of molecular building block that self-assembles by ordered arrangement *via* non-covalent interactions.

Yang et al. (2009) exploited the hydrolytic function of MMP-9 to converse peptide precursors into gelators that could self-assemble into microstructured hydrogels. The designed precursors consisted of short peptides with the amino acid sequence of FFFFCGLDD. The FFFF peptide served as an effective gel factor with a critical gelation concentration of 0.4% (w:v) in water. The cleavage site for MMP-9 is theoretically between G and L. The integrated LDD sequence increased the aqueous solubility of the precursor. The hydrogel was formed after the precursors were incubated with MMP-9 for 45 min, and 2-h incubation brought about 52.8% of FFFFCGLDD, 31.8% of FFFFCG, and 15.4% of FFFF, indicating that MMP-9 could cause the hydrolysis of the FFFFCGLDD peptide and subsequent hydrogel formation *via* the intermolecular non-covalent interactions between the peptide hydrolyzates.

Soellner and colleagues developed a tunable protease-responsive precursor containing three parts: (1) a solubilizing factor, PEG_4_-_*D*_R_*D*_R_*D*_SP composed of oligo(ethylene glycol), three D-type amino acids with proteolytic resistance and a proline; (2) a gelation factor, PABA-F_5_Phe-Phe-NH_2_ (PABA: *p*-aminobenzoic acid, F_5_Phe: pentafluorophenylalanine); and (3) a protease-recognizing tetrapeptide that links the *C*-terminus of the solubilizing factor and the *N*-terminus of the gelation factor (Bremmer et al., 2012). For different proteases, corresponding specific precursors were synthesized by changing the tetrapeptide linkers. The LTPR, AAPF, and DAFE tetrapeptides were designed for thrombin, chymotrypsin, and GLU-C, respectively. After adding the corresponding protease to the precursor solution, the hydrogel formation was observed within hours. Such protease-triggered self-assembly system was suitable for most serine proteases, resulting in no residue left on the *N*-terminus. However, for matrix protease, specific residues are needed on both sides of the cleavable bond, and the leftover residues connecting to the end of the gelation factor might significantly impede the gelation process. Accordingly, they proposed a dual-enzyme-catalyzed self-assembly strategy to avoid the negative effect of the remaining residues on gelation using the matrix protease alone (Bremmer et al., 2014). The precursor was synthesized by introducing the MMP-9-degradable GPKGLKGA peptide linker. MMP-9 and aminopeptidase M (AP-M) were used simultaneously to trigger the assembly of the precursor. AP-M could indiscriminately cleave the remaining amino acid residues from the termini of peptides (Drag et al., 2010) after the MMP-9-mediated peptide degradation, thereby supporting efficient gelation and achieving stable hydrogel.

### Assembly by Bond-Forming Reactions

Proteases have been validated able to exert reverse hydrolysis under non-physiological conditions, such as in organic medium (Xu et al., 2013; Zhu et al., 2018) and at solid/liquid interface (Ulijn et al., 2002). Integrating protease-catalyzed peptide condensation with self-assembly is an appealing approach to shift hydrolysis toward condensation in aqueous medium. Thermolysin and α-chymotrypsin are the mostly used proteases for bond-forming reactions. Ulijn and coworkers first reported the application of protease to selectively instruct assembly of short peptides *via* reverse hydrolysis in 2006 (Toledano et al., 2006), and further characterized this protease-assisted self-assembly under thermodynamic control (Williams et al., 2009). Thermolysin, an endo-protease, was chosen as a model protease and utilized in the reverse hydrolysis reactions, which has a substrate preference for hydrophobic/aromatic residues on the *N*-terminal peptide bonds. Under the catalysis of thermolysin, two non-gelling precursors, *N*-(fluorenylmethoxycarbonyl) (Fmoc)-capped non-polar amino acids (Fmoc-G, Fmoc-L, Fmoc-F, or Fmoc-T) and nucleophiles (GG, FF, LL, or L-OMe, F-OMe esters), resulted in synthesis of Fmoc-peptide gelators and subsequent assembly of nanofibrous structures driven by π–π stacking. The assembled nanofibers with a diameter of ∼10–20 nm became physically entangled and spontaneously formed hydrogel above a critical concentration. It is worth noting that the free energy variation involved in the peptide self-assembly is enough to reverse the hydrolytic reaction, although the gelator synthesis is not thermodynamically favored in itself. Self-correction, component-selection, and spatiotemporal confinement of this reversible self-assembly under thermodynamic control was investigated. Incubating both L-OMe and F-OMe with Fmoc-T simultaneously in the presence of protease generated Fmoc-TF-OMe as the main product with a yield of 82%. Furthermore, the production of Fmoc-TF-OMe prevailed in the reaction, although Fmoc-T was pre-incubated with L-OMe followed by adding of F-OMe. This protease-instructed self-assembly process was validated to be equilibrium-driven and completely reversible, which rendered self-correction of assembly defects in a spatially confined manner.

Thermodynamically and kinetically controlled techniques have been exploited for the protease-catalyzed peptide synthesis. In general, the reaction controlled by kinetics has faster rates than that controlled by thermodynamics. The peptide containing an activated ester as an acyl donor is often required for the kinetically controlled syntheses (Salam et al., 2005). Gross and colleagues reported an α-chymotrypsin-triggered hydrogelation *via* self-assembly of alternating peptide under kinetic control (Qin et al., 2013). The gel formation was observed in the KL-OEt solution (pH 8.5) within less than 10 s after addition of enzyme. The oligomerization of the “dipeptide lego” manipulated by chymotrypsin resulted in mixed chain oligomers (KL)_*x*_ with the average polymerized product (KL)_4.7_, which self-assembled into the β-sheet structures and triggered a sol–gel transition. Chymotrypsin immobilized on the polyethyleneimine/tannic acid membrane generated the mixed product [(KL)_*x*_, *x* = 2–7], which self-assembled to form an entangled nanofibrillar network at the interface (Vigiercarriere et al., 2017). For this enzyme immobilization approach, there was a lag time prior to the beginning of self-assembly, which could be tuned by changing the surface density of chymotrypsin and the concentration of KL-OEt.

Pappas et al. (2015) also reported a transient hydrogel based on the peptide nanofiber assembly that was kinetically controlled but thermodynamically unfavored under certain conditions (Figure 1B). The DF-OMe precursor could react with either F-NH_2_ or Y-NH_2_ to achieve the tripeptide amide gelators, DFF-NH_2_ or DFY-NH_2_ through α-chymotrypsin-catalyzed transacylation. When the concentration of the synthesized tripeptide amide gelators was temporarily higher than the critical gelation concentration, a transient gelation initiated, although the amide hydrolysis was thermodynamically favored. The nanostructure that was formed by the gelation collapsed until the competing hydrolysis was dominant. More importantly, during this enzyme-catalyzed synthesis process, the main product was the kinetically favored DFY-NH_2_ rather than the thermodynamically favored and more stable DFF-NH_2_, suggesting that the kinetic control was dominant within this transient formation of nanostructures. Conte et al. (2018) further significantly increased the lifetime of the transiently formed hydrogel in a non-equilibrium system by immobilizing α-chymotrypsin on magnetic nanoparticles. The percentage of gelator remained as high as 80% after 1 month using the immobilized enzyme, while that dropped to 10% after 72 h using the free enzyme. Upon an external magnetic field, nearly all the tripeptide gelators assembled into the nanofibers due to a local high concentration. The immobilization of enzyme also markedly hampered its hydrolytic activity on the tripeptide-based building blocks. Accordingly, the degradation of the self-assembled fibers was noticeably delayed and the lifetime of the hydrogel was dramatically prolonged.

## Biomedical Applications

The self-assembly of small-molecule peptides leads to the formation of entangled nanofibers on the microscopic level which can entrap water to form hydrogel on the macroscopic level. The hydrogel possesses unique characteristics, such as high water content, highly porous architecture, tunable flexibility, and structural similarity to natural extracellular matrices, which has been considered as an ideal reservoir for drug delivery as well as a superior scaffold for tissue engineering (Jiang et al., 2018; Li et al., 2019; Gao et al., 2020). Moreover, the *in situ* self-assembly of nanofibers in response to endogenous proteases in the tissue or cellular microenvironments as a potential strategy achieves site-specific retention and even generates selective cytotoxicity toward cancer cells.

Jiang et al. (2017) reported a substrate-selective protease-catalysis strategy to trigger formation of a short peptide-based hydrogel for localized delivery of protein therapeutics (Figure 1C). WQ9-2, a metalloprotease, was used as a model protease in the reverse hydrolytic reaction (Xu et al., 2013), which could catalyze the Fmoc-F and FF-Dopa precursors to converse into the Fmoc-FFF-Dopa gelator that self-assembled into the nanofibrous hydrogel. However, WQ9-2 would degrade the encapsulated proteins rapidly, such as tumor necrosis factor-related apoptosis-inducing ligand (TRAIL) and hirudin. To solve this issue, WQ9-2 was encapsulated into a single-protein nanocapsule by weaving a polymeric shell around. The nanocapsule allowed permeation of the small-molecule precursors and maintained the catalytic activity of WQ9-2, but suppressed the proteolytic effect of WQ9-2 on the loaded macromolecular proteins due to spatial hindrance provided by the polymeric sheath. The TRAIL and hirudin co-loaded hydrogel using the WQ9-2 nanocapsule-mediated assembly strategy exhibited synergistic tumor-inhibiting effects on the breast tumor mouse model after a single-dose intratumoral injection. Jiang et al. (2018) also used the WQ9-2-manipulated oligopeptide hydrogel for topical delivery of paclitaxel that was loaded in cell-penetrating peptide (CPP)-modified transfersomes (PTX-CTs). The transfersome was composed of phospholipid and surfactants such as Tween 80 and sodium deoxycholate. The flexible and deformable transfersome could efficiently penetrate the stratum coreum through the intercellular gaps into the epidermis. Meanwhile, the incorporated surfactants and CPP also play significant roles in enhancing the transdermal efficiency of the transfersome. The PTX-CTs embedded hydrogel (PTX-CTs/Gel) was topically painted as a drug depot, which enhanced the skin retention of PTX-CTs. The released PTX-CTs from the hydrogel promoted the skin and tumor penetration of PTX and led to increased cytotoxicity against melanoma cells. PTX-CTs/Gel served as an adjuvant treatment combined with the systemic chemotherapy to efficiently inhibit the melanoma growth on the mouse model.

Kalafatovic et al. (2016) proposed the *in situ* self-assembly strategy manipulated by MMP-9 to increase the cytotoxic activity of chemotherapeutic drugs. Two MMP-9-cleavable peptides, PhAc-FFAGLDD and GFFLGLDD, were synthesized, both of which could assemble into micelles to encapsulate the small-molecule anticancer drug, doxorubicin. Interestingly, they found that PhAc-FFAGLDD and GFFLGLDD converted mainly into PhAc-FFAGL and GFFLGL under the catalysis of MMP-9, respectively, which was different from the expected proteolytic cleavage site between G and L. After intravenous injection, the peptide-based micelles accumulated in the tumor tissue. MMP-9 that was highly expressed in the tumor microenvironment could hydrolyze the micelles, leading to a micelle-to-nanofiber transformation. The formed nanofibers showed prolonged intratumor retention than the original micelles, and acted as a drug reservoir with sustainable release of doxorubicin, resulting in higher effect on inhibiting tumor growth of the triple negative breast tumor mouse model. In contrast, the doxorubicin-loaded D-type peptide-assembled micelles could not be degraded by MMP-9 and converse into nanofibers, which had no effect on tumor growth inhibition.

Tanaka et al. (2015) synthesized a palmitoyl-modified peptide, C16-GGGHGPLGLARK for the *in situ* self-assembly in response to MMP-7 (Figure 1D). In the structure of the lipid peptide, the C16 alkyl chain served as a hydrophobic moiety; GGGH provided hydrogen bonds; PLGL was an MMP-7-degradable moiety with the cleavage site between G and L; RK was incorporated as cationic heads. In the absence of MMP-7, the lipid peptide precursor could not be gelled due to the electrostatic repulsion between the cationic RK moieties, while it was converted into the C16-GGGHGPLG gelator and formed nanofibers in the presence of MMP-7. The *in vitro* cytotoxicity studies showed that treatment with C16-GGGHGPLGLARK caused high cytotoxic activity against five different cancer cell models, such as MCF-7, SKBR3, MIAPaCaII, HeLa, and A431 cells, but low cytotoxicity on normal cell models, such as MvE and PE cells. The cytotoxicity was mainly attributed to the vital stress on the cancer cells caused by the intracellularly formed nanofibers. Moreover, the cytotoxicity induced by the peptide was positively correlated with the expression level of MMP-7 within the cancer cells.

In addition to cancer therapy, Carlini et al. (2019) reported the *in situ* peptide assembly approach as a promising hydrogel delivery strategy for cardiovascular disease (Figure 1E). Two cyclic peptide precursors, KLDL_*Cyclic*_ (NH_2_-C(KLDL)_3_PLGLAGC-CONH_2_) and KFDF_*Cyclic*_ (NH_2_-C(KFDF)_3_PLGLAGC-CONH_2_), were developed. Under the catalysis of MMP-9, the precursor transformed into a linear peptide with self-assembly ability, and subsequently formed a hydrogel. The precursor solution with favorable fluidity could flow freely through a syringe without clogging and cytotoxicity, and gelate at the site of myocardial infarction with a high level of MMP-9 expression on the rat model.

Wang et al. (2020) used thermolysin to trigger the self-assembly of short peptides in the pores of anodic aluminum oxide (AAO) template for cell culture. The AAO templates with highly-ordered porous structures, high-density pore distribution, and tunable channels offered an ideal confined environment for precise control of peptide assembly. Fmoc-threonine/tyrosine (Fmoc-T/Y) and amide-modified leucine (L-NH_2_) were condensed by thermolysin to form Fmoc-T/YL-NH_2_. The formed Fmoc-T/YL-NH_2_ molecules could self-assemble into fibers that are perpendicular to the channel walls of the AAO templates. The length of fibers was only determined by the pore size of templates rather than the depth. Meanwhile, the precursor concentration had notable effect on the formation of fibers. Higher concentration resulted in denser and thicker fiber networks. The cell morphology and extension of the fibroblasts cultured on the template were obviously affected by the assembled peptide fibers, but no significant impact on the cell viability was observed.

Williams et al. (2011) reported a protease-instructed peptide hydrogel for localized delivery of laminin, an extracellular matrix protein to the damaged tissue on a zebrafish model of muscular dystrophy. The hydrogel was formed by the immobilized thermolysin-mediated self-assembly of Fmoc-LLL *via* π-stacking interactions, which was loaded with laminin and locally injected into a dystrophic zebrafish model. The laminin-loaded hydrogel showed enhanced stability and prolonged persistence within the target tissue *in vivo*, and was demonstrated to be a promising drug delivery system for treatment of diseases caused by structural failure of the extracellular matrix.

## Conclusion and Future Perspectives

Considerable efforts have been increasingly made to pursue biomimic strategies to precisely regulate and control the bottom-up nanofabrication process for production of ordered architecture. We and other researchers have identified that the protease-manipulated self-assembly approach may be suitable for well-defined nanofabrication under thermodynamic or kinetic control. Increasing attention on kinetic regulation allows for creating controllable and tunable structures which do not represent thermodynamic equilibrium. Protease screening and engineering as well as rational design of substrate repertoire can be tuned to generate asymmetric, dynamic, and multicomponent structures with superb functions. The macroscale peptide-based hydrogel formed by the protease-instructed self-assembly can serve as reservoir for drug delivery as well as scaffold for tissue engineering. The *in situ* self-assembly of peptides in response to endogenous protease in the physiological or pathological microenvironment can be used to achieve site-specific retention in the targeted tissue and even to induce cytotoxicity toward cancer cells. However, several challenges still remain in spite of significant achievements in the field of protease-instructed peptide assemblies. The macroscale hydrogel formed by the short peptide often has a relatively lower mechanical strength. Moreover, the *in vivo* behaviors of the peptide assemblies should be comprehensively evaluated and investigated prior to clinical translation, such as metabolism, excretion, and safety, although the peptide-based materials are regarded to possess good biocompatibility and biodegradability. In addition to the peptide self-assembly, enzymes have also been widely exploited to control the assembly of polymers (Teixeira et al., 2012), deoxyribonucleic acids (Park et al., 2009), and nanoparticles (Yang et al., 2018). In a word, the protease-instructed self-assembly supports abundant production of unique nanostructures with desired functions for potential applications in bionanotechnology and biomedicine.

## Author Contributions

TJ, BH, and RM conceived the idea and organized this mini review. All authors contributed to writing, editing, and literature review.

## Conflict of Interest

The authors declare that the research was conducted in the absence of any commercial or financial relationships that could be construed as a potential conflict of interest.
